# Application of network pharmacology and molecular docking to elucidate the potential mechanism of *Eucommia ulmoides*-*Radix Achyranthis Bidentatae* against osteoarthritis

**DOI:** 10.1186/s13040-020-00221-y

**Published:** 2020-08-28

**Authors:** Gong-hui Jian, Bing-zhu Su, Wen-jia Zhou, Hui Xiong

**Affiliations:** Hunan University of Chinese Medicine, Changsha, Hunan Province People’s Republic of China

**Keywords:** Osteoarthritis, Network pharmacology, Traditional Chinese medicine, *Eucommia ulmoides*, *Radix Achyranthis Bidentatae*, Molecular docking

## Abstract

**Background:**

Osteoarthritis is a disabling disease, which seriously affects the quality of life of patients. Increasing evidence has indicated that Chinese herbal medicine including *Eucommia ulmoides* (EU) and *Radix Achyranthis Bidentatae* (RAB) have potential in the treatment of osteoarthritis, and this is associated with their multi-target and multi-link action characteristics. Although their potential anti-arthritic activity has been reported, the exact mechanism of EU-RAB action in osteoarthritis remains unexplored. Therefore, this study explores the mechanism of EU-RAB against osteoarthritis using network pharmacology and molecular docking technology.

**Methods:**

Public databases including TCMSP、BATMAN-TCM、OMIM and Genecards were used to predict the bioactive ingredients and putative targets of EU-RAB against osteoarthritis. Enrichment analysis was performed to expound the biological functions and associated pathways of the hub targets. Cytoscape software was used to construct a “compounds-targets-pathways” network for elucidating the comprehensive molecular mechanism of EU-RAB against osteoarthritis. Molecular docking was used to verify the correlation between the main active ingredients and hub targets.

**Results:**

Network pharmacological analysis of EU-RAB in the treatment of osteoarthritis, identified 50 active ingredients including quercetin, kaempferol, wogonin, and baicalein with important biological effect. A total of 68 key targets were screened, including IL-6, EGFR, MAPK8, etc., and they were found to be enriched in a series of signaling pathways, such as apoptosis, TNF, MAPK, PI3K/AKT, and IL-17 signaling pathways. Moreover, molecular docking analysis showed that the main ingredients were tightly bound to the core targets, further confirming the anti-arthritic effects.

**Conclusion:**

Based on network pharmacology and molecular docking analysis, the present study provides insights into the potential mechanism of EU-RAB in osteoarthritis after successfully screening for associated key target genes and signaling pathways. These findings further provide a theoretical basis for further pharmacological research into the potential mechanism of EU-RAB in osteoarthritis.

## Introduction

Osteoarthritis (OA) is characterized by progressive degeneration and wear of cartilage as well as subchondral osteosclerosis, pain, swelling, and stiffness in the joints. Globally, osteoarthritis affects approximately 250 million people [1]. Chronic pain and disability associated with OA can lead to anxiety, depression, and suicidal emotion [2]. However, long-term use of non-steroidal anti-inflammatory drugs in patients with OA is associated with gastrointestinal and cardiovascular side-effects. Recent therapies in regenerative medicine including stem cell and platelet-rich plasma therapy are commonly used in the short-term treatment of localized pains such as joint pain [3]. Therefore, there is an urgent need for systematic and effective clinical treatment approaches to reduce chronic pain and improve the quality of life in patients with OA.

In the field of Traditional Chinese Medicine (TCM), OA is referred to as Arthralgia Syndrome, which originated from the ancient book “Huangdi Neijing”. Valuable research over the years has explored the role of TCM in the treatment of OA, via the mechanisms of anti-inflammation, anti-apoptosis, and anti-catabolism [4]. *Eucommia ulmoides* (EU, Chinese name: Duzhong) and *Radix Achyranthis Bidentatae* (RAB, Chinese name: Niuxi), are natural herbs reported to nourish kidney and strengthen the bone and are recorded in the classic of Herbal medicine Shennong Ben Cao Jing, which is one of China’s earliest pharmaceutical works [5, 6]. Research shows that the EU is currently the most widely used traditional Chinese medicine in the treatment of OA, while RAB is ranked third [7]. Moreover, both are often used and recognized as classical pair drugs playing a synergistic role in anti-osteoarthritis [8, 9]. Xie et al. showed that EU extract suppresses the activity of the PI3K/Akt pathway to mitigate the release of inflammatory factors which play a significant role in anti-osteoarthritis [10]. Zhang et al. demonstrated that EU extract inhibited bone loss and maintained metabolic balance [11]. Ecdysterone and saponins extracted from RAB were reported to protect chondrocytes through anti-apoptosis and anti-inflammation effects [12, 13]. Weng et al. revealed the positive effect of *Achyranthes bidentata* polysaccharides on chondrocytes proliferation [14]. Therefore, it is expected that a mixture of EU and RAB is capable of having potent anti-osteoarthritis effects. However, the potential pharmacological mechanisms of action of EU-RAB and their interaction with osteoarthritis-related targets and pathways remain unknown and need to be further explored.

**Fig. 1:**
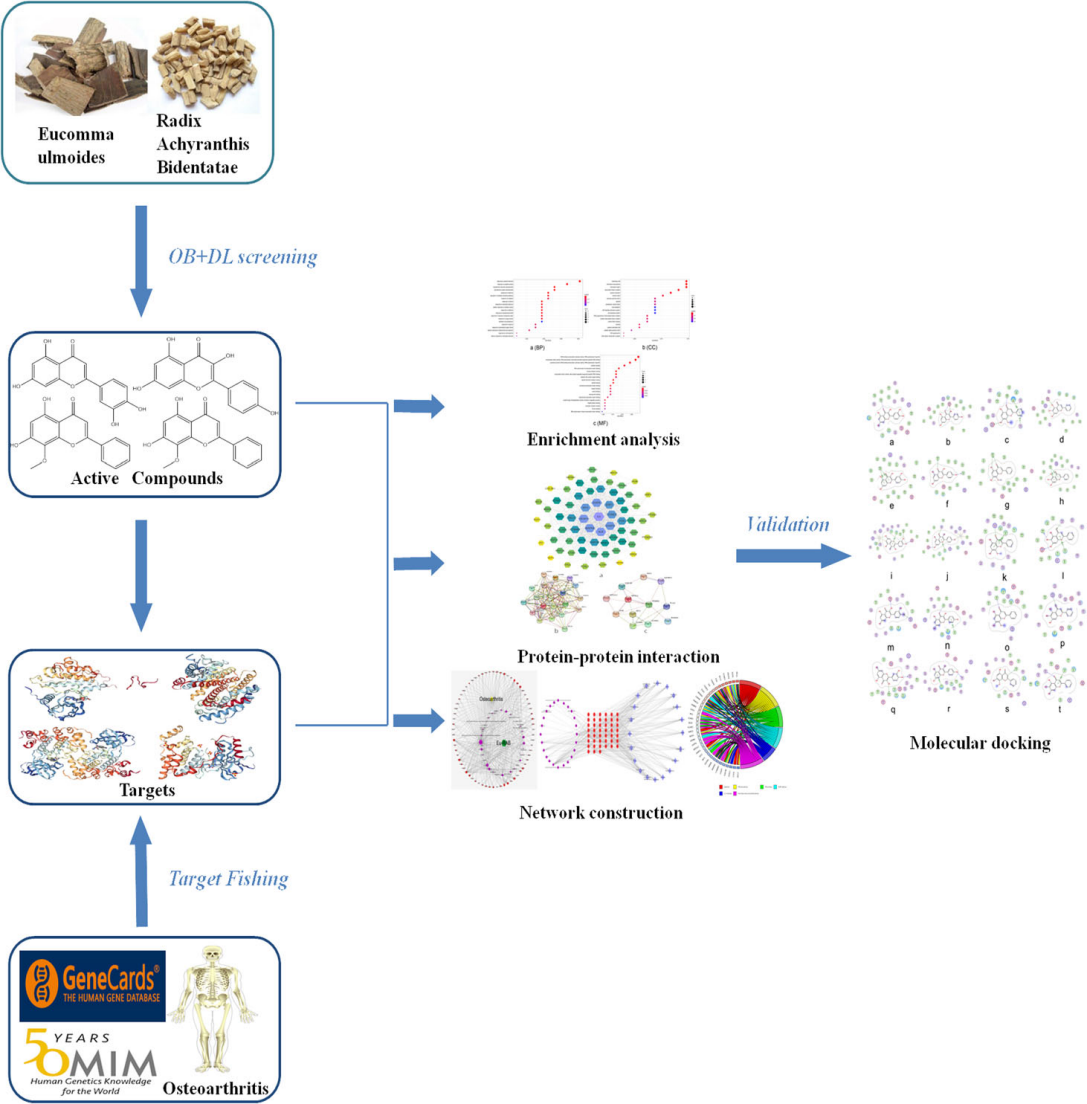
Schematic diagram for identifying the mechanism of *Eucommia ulmoides*-*Radix Achyranthis Bidentatae* anti-Osteoarthritis by network pharmacology analysis

TCM has a complex mechanism of action and multi-pathway interactions. Over the past few decades, Chinese medicine was regarded as empirical medicine, which was neglected due to a lack of modernized research approaches in related disciplines such as molecular biology, pharmacology, and bioinformatics, etc. [15]. Network pharmacology is a novel method that combines computer science with medicine. Based on” multi-gene, multi-target, multi-pathway” interaction network, network pharmacology combined with TCM provides an in-depth understanding of the active ingredients in Chinese herbs [16]. Besides, this also provides potent evidence into biological targets and the underlying mechanism of action of TCM in the treatment of various diseases [17]. This study seeks to elucidate the molecular targets and underlying mechanism of action of EU-RAB in osteoarthritis using network pharmacology and molecular docking (Fig. 1).

## Methods

### Identification of active ingredients in EU-RAB

The compounds-related data of EU and RAB were retrieved from Traditional Chinese Medicine Systems Pharmacology (TCMSP) database (http://lsp.nwu.edu.cn/tcmsp.php), which is a unique platform for the analysis of active ingredients of Chinese herbal medicine and their interaction in specific diseases [18], and the Bioinformatics Analysis Tool for Molecular mechanism of Traditional Chinese Medicine (BATMAN-TCM) database (http://bionet.ncpsb.org/batman-tcm/), which is the first online bioinformatics analysis tool for screening of the molecular mechanism of TCM [19]. Oral bioavailability (OB) and drug-likeness (DL) are important pharmacokinetic parameters used to identify and screen active ingredients based on the ADME (absorption, distribution, metabolism, and excretion) model [20].

OB represents the relative amount and rate at which the drug is orally absorbed into the body circulation. Most TCM compounds are difficult to reach specific tissues and exert certain pharmacological effects, mainly due to poor OB [21]. In the TCMSP database, a widespread computer prediction model OBioavail 1.1 [22] has been used to calculate the OB value of compounds. Based on the recommendation in the TCMSP database and previous studies [23], compounds with OB ≥ 30% are considered to have reliable pharmacological activity.

DL is a concept that is based on the physical and chemical properties and molecular structure of existing drugs and is used to evaluate whether a compound meets the conditions to become a new drug. In this study, the Tanimoto coefficient [24] was used to calculate the DL (see Eq. (1)) of each compound in EU-RAB.

1 $$ \mathrm{F}\left(\mathrm{X},\mathrm{Y}\right)=\frac{XY}{\left(\left|X\right|2+\left|Y\right|2- XY\right)} $$

In this equation, X represents the molecular descriptors of a new ingredient, and Y represents the average molecular properties of 6511 molecules in the DrugBank database (http://www.drugbank.ca). The DL threshold is determined based on the average DL value of the Drugbank being 0.18.

In the database, we obtained the corresponding ingredients by inputting the names of the herbal medicine and then selecting the active ingredients for further analysis using the criteria: OB ≥ 30% and DL ≥ 0.18.

### The putative targets of EU-RAB

TCMSP database not only allows users to analyze to identify active compounds of Chinese herbal medicine but to also search for related genes targets of the active compounds. After obtaining the active ingredients by limiting OB and DL, the targets of each ingredient were collected and the repeating parts were removed to obtain the EU-RAB related targets. The species of target proteins were set to “*Homo sapiens*” and the whole gene information including gene name, gene ID, gene symbol was obtained using Uniprot protein sequence resource (https://www.uniprot.org).

### Related targets of osteoarthritis and prediction of potential targets of EU-RAB against osteoarthritis

Osteoarthritis targets were acquired from two databases. GeneCards (https://www.genecards.org/, ver. 4.9.0) database is a human gene online analysis platform which is based on abundant gene resources, it integrates and predicts gene targets associated with diseases and also used to conduct Gene-Centered “OMIC” research [25]. OMIM (http://www.omim.org/, updated February 19, 2019) database is centered on the connection between the gene and phenotype, maintained timely updating [26]. The keyword “osteoarthritis” was searched in the two databases to obtain the disease-targets; then, the overlapping part after integrating the disease-targets and drug-targets were the potential targets of EU-RAB against osteoarthritis.

### Protein-protein interaction

Protein-protein interaction (PPI) analysis helped in the identification of key targets belonging to both EU-RAB and OA, and also highlight the related hub genes of EU-RAB anti-OA. The active ingredient of EU-RAB relevant to OA targets was retrieved via the STRING (https://string-db.org/,ver. 11.0) database, mined the key regulatory genes. The potential targets were inputted to “Multiple proteins” in STRING and the organism was limited to “*Homo sapiens*”. A confidence score with correlation degree ≥0.400 was set using the default threshold, and this enabled the enhanced interaction between targets while ensuring a positive rate and extracted the PPI network after removing the discrete genes.

In the PPI network, many closely interconnected regions were defined as clusters or topology modules, which were constructed by each node and edge as well as made to reflect on related molecular biological functions and core protein processes [27]. Clusters can be obtained using MCODE, a plug-in of Cytoscape 3.7.2. Each cluster of proteins was inputted, to reproduce the corresponding cluster map and predict functional links between proteins.

### Enrichment analysis

Gene ontology (GO) and Kyoto Encyclopedia of Genes and Genomes (KEGG) pathway enrichment analyses were conducted to explore the core mechanism of action and biological pathways associated with the drug. After the PPI network was successfully constructed, hub co-targets were imported into R software and the clusterprofile, a bio-conductor package, was used to perform the GO and KEGG enrichment analysis, to obtain the biological processes, cellular components, molecular function and key signaling pathways. Only functional annotations with enrichment *p* values smaller than 0.05 were chosen for further analysis.

### Network construction

Network construction was performed using Cytoscape (ver. 3.7.2), a network visualization software to intuitively display the compound-target-pathway relationship between EU-RAB and OA, including 3 networks, namely: 1) compound-target network of EU-RAB; 2) potential compounds-potential targets network of EU-RAB for anti-osteoarthritis; 3) potential compounds-potential targets–pathways network of EU-RAB for anti-osteoarthritis.

### Verification of compound-target interaction through molecular docking

Molecular docking technology was based on the “Key-Lock principle” to reflect the small molecule ligands-protein receptors association. Further, the best binding mode and site were searched based on the conformation change and energy matching process. In this study, the Molecular Operating Environment (MOE) (v2015.10) was performed to validate compound-protein target interaction. The crystal structure of the protein targets was downloaded from the RCSB PDB database (http://www.rcsb.org/). The imported crystal structure was imported into MOE for use in constructing the receptor protein grid, which was associated with the processes of protonation, removal of water molecules and repetitive structure, structure preparation, and energy minimization. The receptor grid construction was used to select the binding site with ligands. Lastly, the three-dimensional compound structure was edited in ChemBioDraw software, imported into MOE, and docked with protein construct to obtain the graphical results of molecular docking.

## Results

### Active compounds of EU-RAB

A total of 433 EU-RAB compounds were retrieved from the TCMSP and BATMAN-TCM database. Based on the screening criteria, OB ≥ 30% and DL ≥ 0.18, 31 active ingredients in the EU, and 22 active ingredients in RAB were obtained. There were 50 active ingredients after deleting repetitions, such as quercetin, kaempferol, and wogonin. The details are shown in the Supplementary file, Table S1.

### Putative targets of EU-RAB

In this study, 102 putative targets for the EU, and 100 putative targets for RAB were identified. However, most of the targets were repetitive, revealing that the active ingredients of the EU and RAB may possess identical biological effects and achieve a cooperative effect when combined. A total of 110 putative targets of EU-RAB (Supplementary file, Table S2) were obtained by further integrating all the targets.

### Gene targets of OA and potential targets of EU-RAB against OA

A total of 2498 OA related gene targets were retrieved from the OMIM and Genecards databases. Further, 68 putative targets of EU-RAB were obtained after integrating and connecting 110 putative targets of EU-RAB with 2498 OA gene targets. IL-6, EGFR, GSK3β, ESR, etc. are some of the densely related targets identified between EU-RAB and OA, and details are shown in Table 1.

**Table 1:** Potential targets of *Eucommia ulmoides* (EU)-*Radix Achyranthis* *Bidentatae *(RAB) against Osteoarthritis (OA)

N0.	Gene	N0	Gene	N0	Gene	N0	Gene
1	PTGS1	18	NFKBIA	35	CCNB1	52	GSTM1
2	AR	19	POR	36	ALOX5	53	PGR
3	PPARG	20	CASP8	37	GSTP1	54	ESR1
4	F7	21	PRKCA	38	NFE2L2	55	IKBKB
5	RELA	22	HIF1A	39	PARP1	56	MAPK8
6	EGFR	23	ERBB2	40	AHR	57	ALB
7	VEGFA	24	CYP3A4	41	COL3A1	58	CTNNB1
8	CCND1	25	CAV1	42	NR1I3	59	CASP7
9	BCL2	26	MYC	43	HSF1	60	GSK3B
10	FOS	27	CYP1A1	44	CRP	61	DRD2
11	EIF6	28	ICAM1	45	RUNX2	62	NR3C1
12	CASP9	29	SELE	46	CTSD	63	ESR2
13	PLAU	30	VCAM1	47	IGFBP3	64	FOSL1
14	IL6	31	PTGER3	48	IGF2	65	CYCS
15	CASP3	32	BIRC5	49	IRF1	66	NOX5
16	TP63	33	NOS3	50	PON1	67	TEP1
17	ELK1	34	HSPB1	51	DIO1	68	MCL1

### Enrichment analysis

**Fig. 2:**
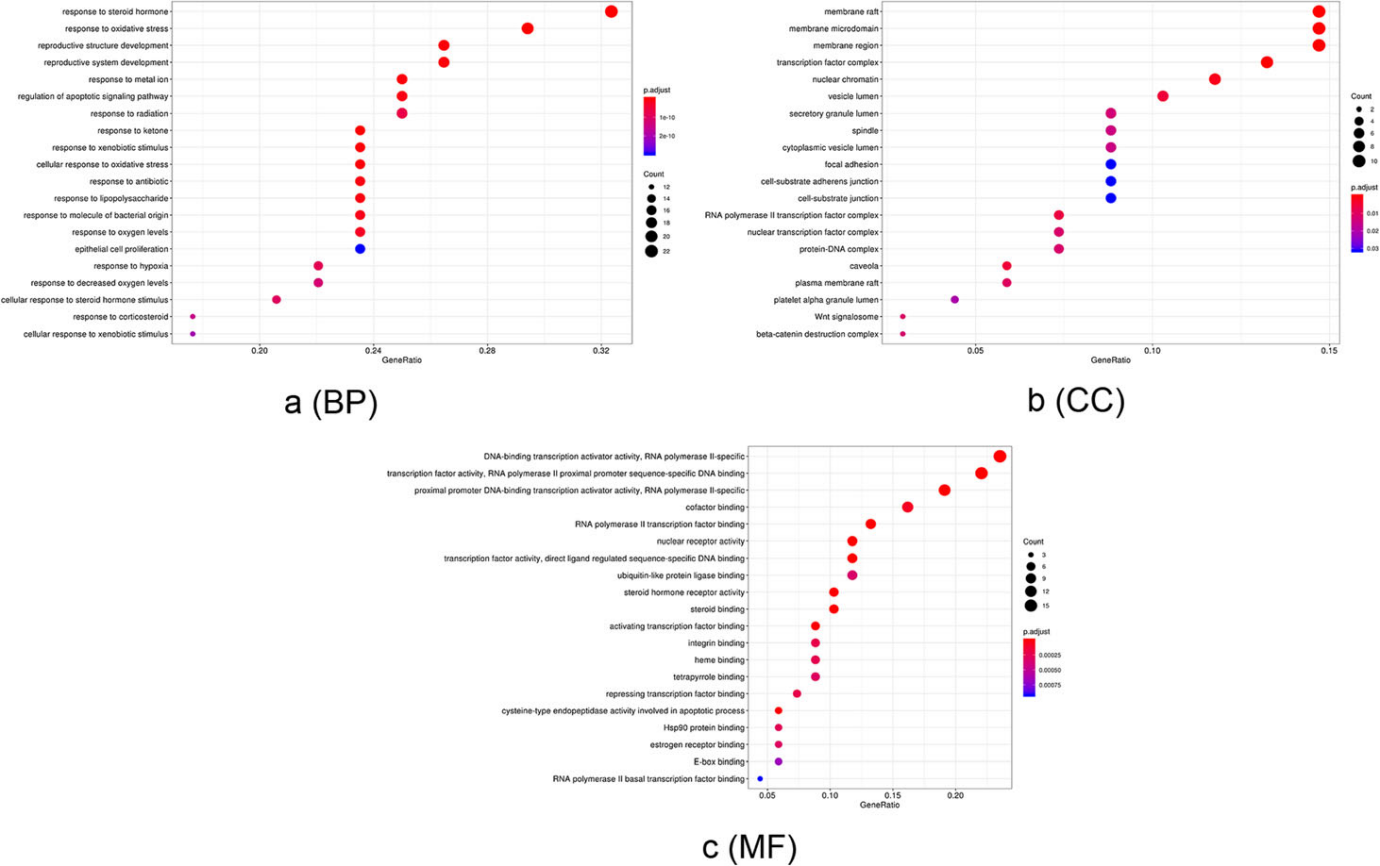
The GO enrichment analysis of genes of *Eucommia ulmoides*-*Radix Achyranthis Bidentatae* against Osteoarthritis. The ontology covered three domains: biological process(**a**), cellular component (**b**), and molecular function (**c**). The representative of abscissa GeneRatio, ordinate, size of the dots, and color of the dots represent the degree of GO enrichment analysis

GO and KEGG pathway enrichment analysis contributed to further analysis of cellular localization, biological effect, pathway, and function of the 68 gene targets of EU-RAB anti-OA. In GO analysis, the 68 potential genes were highly enriched in 1272 biological processes (BP), 31 cellular components (CC), and 95 molecular functions (MF) with *p*-value < 0.05. As shown in Fig. 2, response to steroid hormone (*p* = 3.91E^− 17^, FDR = 1.99E^− 17^), regulation of apoptotic signaling pathway (*p* = 1.16E^− 11^, FDR = 5.88E^− 12^), response to oxidative stress (*p* = 6.98E^− 14^, FDR = 3.55E^− 14^) and cellular response to oxidative stress (*p* = 3.75E^− 12^, FDR = 1.91E^− 12^) were the closely related to OA biological processes. In regard to cellular components, higher enrichment was found in membrane raft (*p* = 9.25E^− 06^, FDR = 6.84E^− 06^), membrane microdomain (*p* = 9.25E^− 06^, FDR = 6.84E^− 06^) and membrane region (*p* = 9.25E^− 06^, FDR = 6.84E^− 06^). The main OA-related terms in molecular functions contained DNA-binding transcription activator activity, RNA polymerase II-specific (*p* = 1.56E^− 09^, FDR = 8.48E^− 10^) and cysteine-type endopeptidase activity involved in apoptotic process (*p* = 9.18E^− 06^, FDR = 5.00E^− 06^) and estrogen receptor binding (*p* = 3.11E^− 04^, FDR = 1.70E^− 04^).

**Fig. 3:**
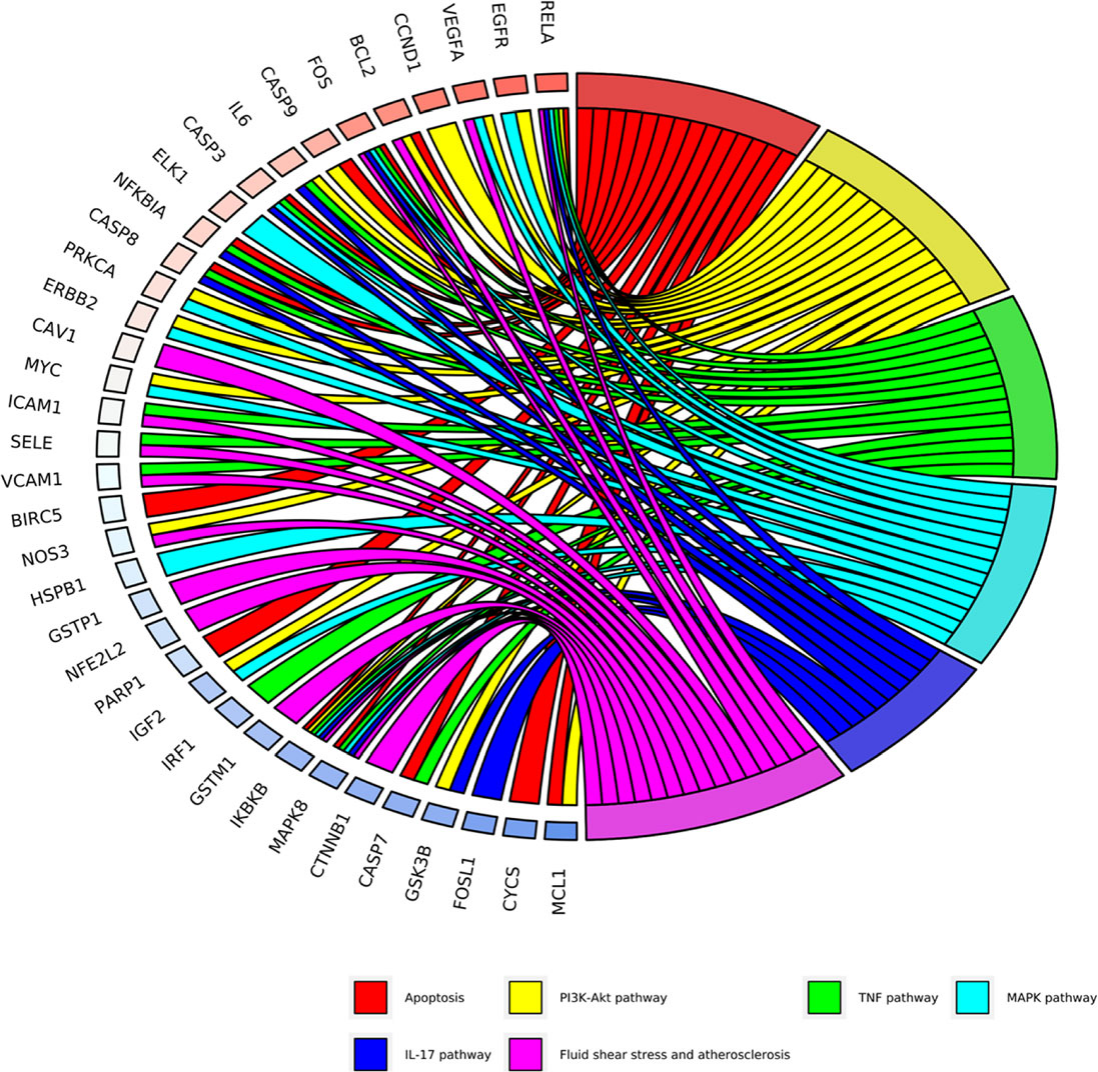
The KEGG enrichment analysis of genes of *Eucommia ulmoides*-*Radix Achyranthis Bidentatae* against Osteoarthritis. Different colors on the right side of the graph represent different signal pathways, and the left side is the core gene with relevance. The more lines in the pathway, the more genes are enriched

KEGG enrichment analysis focused on the anti-osteoarthrits pathways of EU-RAB. Results demonstrated that “Apoptosis”, “TNF signaling pathway”, “PI3K-Akt signaling pathway”, “MAPK signaling pathway” and “IL-17 signaling pathway” were highly associated with the onset and progression of osteoarthritis (Table 2 and Fig. 3). Besides, “Apoptosis” was the pathway found to be the closest and with a high enrichment degree in OA, and mapped 15 gene targets.

**Table 2:** KEGG pathway analysis of *Eucommia ulmoides* (EU)-*Radix Achyranthis* *Bidentatae* (RAB) in treating Osteoarthritis (OA) network

No.	Pathwy ID	Pathway name	*P* value	FDR	Count	Gene name
1	04210	Apoptosis	7.20E-14	2.70E-12	15	RELA,BCL2,FOS,CASP9,CASP3,NFKBIA,CASP8,BIRC5,PARP1,CTSD,IKBKB,MAPK8,CASP7,CYCS,MCL1
2	04151	PI3K-Akt signaling pathway	5.82E-08	2.05E-07	15	RELA,EGFR,VEGFA,CCND1,BCL2,CASP9,IL6,PRKCA,ERBB2,MYC,NOS3,IGF2,IKBKB,GSK3B,MCL1
3	04668	TNF signaling pathway	2.09E-12	1.88E-11	13	RELA,FOS,IL6,CASP3,NFKBIA,CASP8,ICAM1,SELE,VCAM1,IRF1,IKBKB,MAPK8,CASP7
4	04010	MAPK signaling pathway	3.31E-07	8.64E-07	13	RELA,EGFR,VEGFA,FOS,CASP3,ELK1,PRKCA,ERBB2,MYC,HSPB1,IGF2,IKBKB,MAPK8
5	04657	IL-17 signaling pathway	1.99E-09	1.08E-08	10	RELA,FOS,IL6,CASP3,NFKBIA,CASP8,IKBKB,MAPK8,GSK3B,FOSL1

**Table 3:** Virtual docking of four bioactive ingredients from *Eucommia ulmoides* (EU)-*Radix Achyranthis Bidentatae* (RAB) for Osteoarthritis (OA) targets

No	Compound	Structure	Binding Energy /(kcal·mol^−1^)				
			EGFR	ESR1	MAPK8	GSK3β	IKBKB
1	quercetin		−6.9327	−6.8972	− 7.1388	− 6.1622	−7.1583
2	kaempferol		−7.0913	− 6.3109	−6.6991	− 6.1155	−6.5508
3	wogonin		−6.4374	− 6.4663	−6.7080	− 6.1687	−6.9841
4	baicalein		−6.3727	− 6.5548	−6.9782	−5.2388	− 6.3864

### Network construction analysis

#### Potential targets of EU-RAB network analysis

**Fig. 4:**
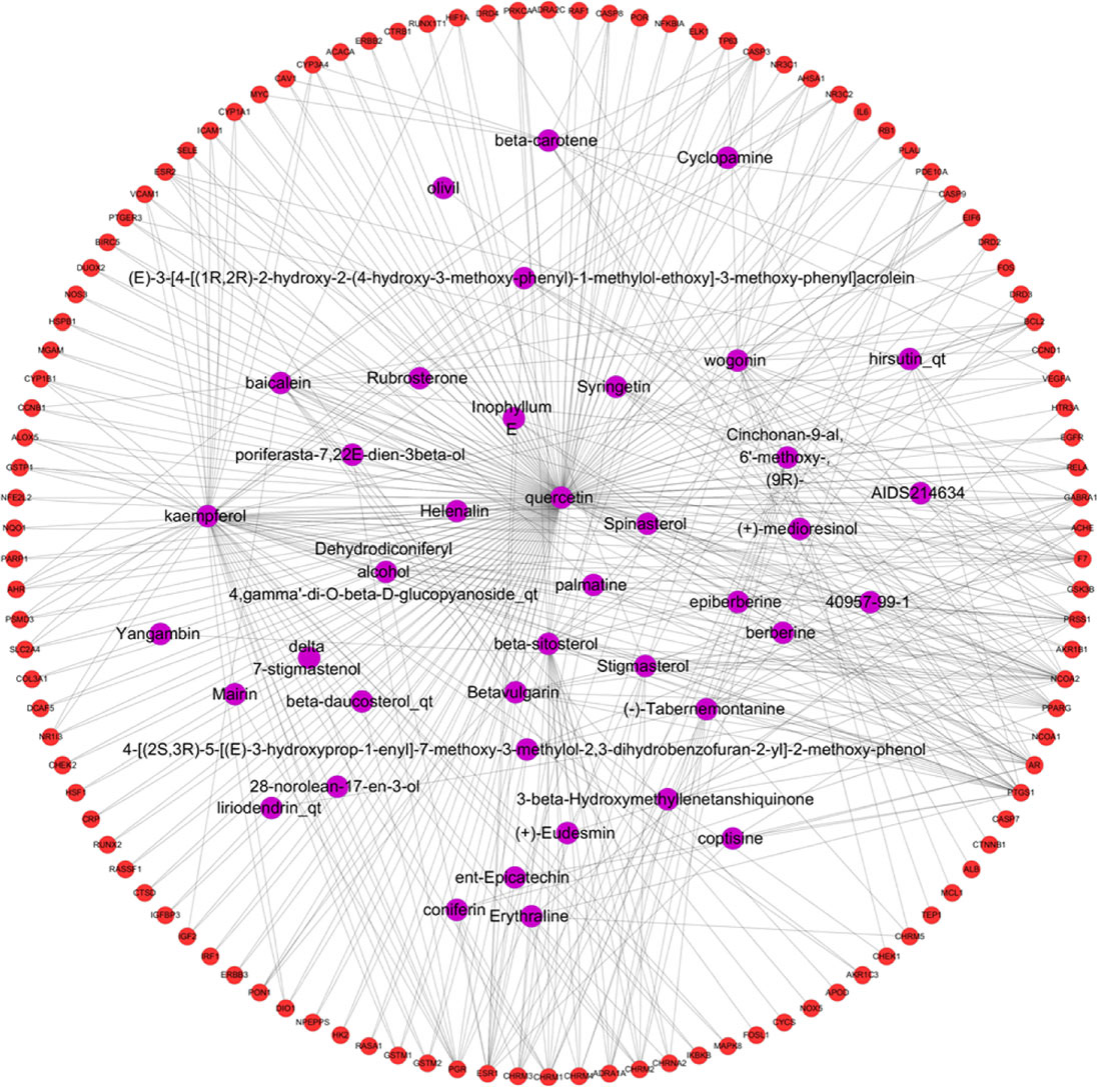
Potential targets network of *Eucommia ulmoides* (EU)-*Radix Achyranthis Bidentatae* (RAB). The potential target network of EU-RAB is constructed using 50 compounds and 110 potential targets. Purple and red cycles represent compound and target respectively. Lines stand for the relation of compounds and target nodes

The network construction of active compound-potential targets of EU-RAB using 50 compounds and 110 targets was based on the “one to multiple”, “multiple to one” links between 150 nodes and 454 edges in this complex network (Fig. 4). The more edges and nodes obtained, the stronger the node interaction, and versa vice. In this network, quercetin (degree = 154), kaempferol (degree = 68), wogonin (degree = 20), and baicalein (degree = 19) were the main active ingredients of EU-RAB for the treatment of various diseases.

#### Construction of a compound-target network of EU-RAB anti-OA

**Fig. 5:**
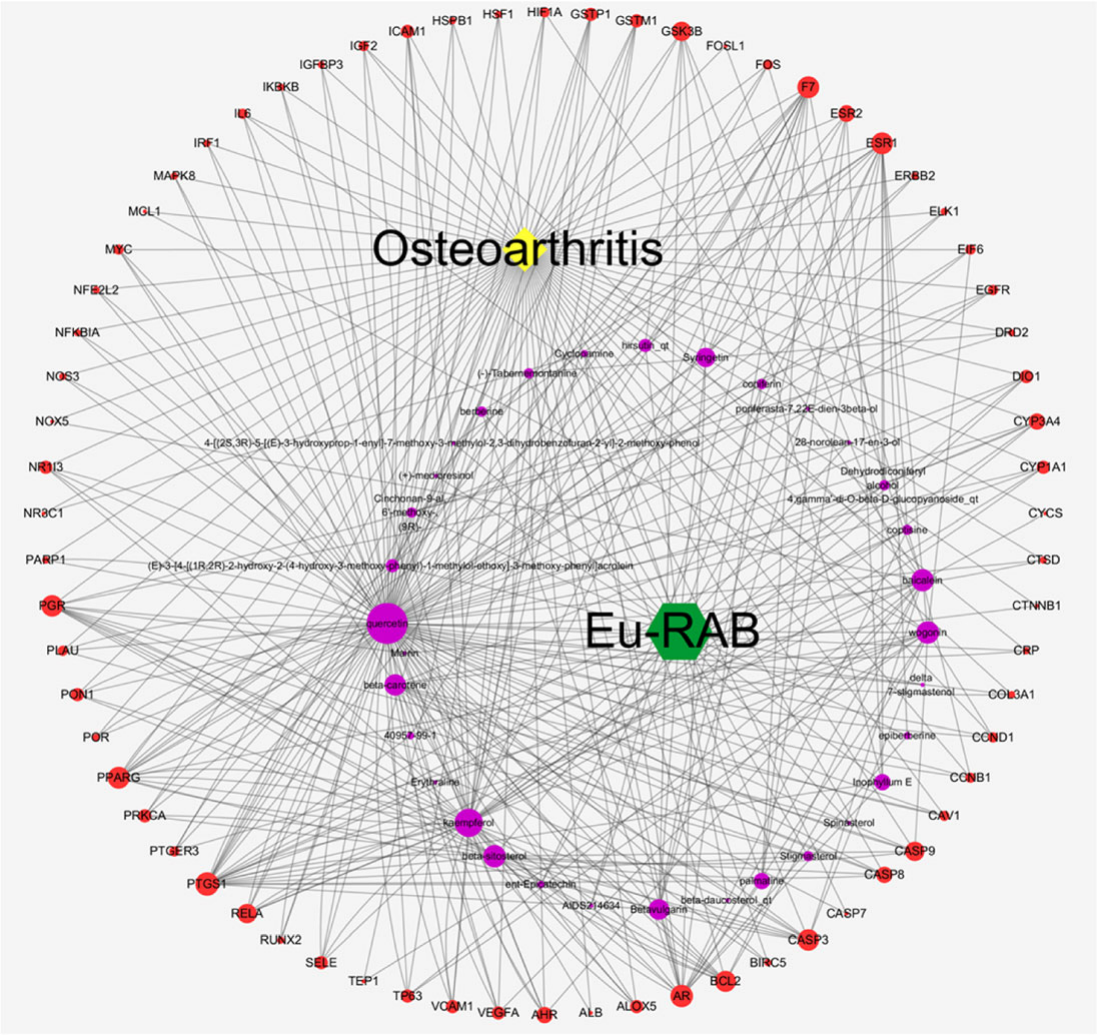
Compound-target network of *Eucommia ulmoides* (EU)-*Radix Achyranthis Bidentatae* (RAB) anti-Osteoarthritis (OA). The Compound-target network of EU-RAB anti-OA is constructed using 50 compounds and 68 co-targets, containing 103 nodes and 379 edges. Purple, red, yellow, and green cycles represent compound, target, osteoarthritis, and EU-RAB, respectively. Lines stand for the relation of compounds and target nodes. In all compounds and targets, the lager of node, the higher degree of constituent

A total of 50 active compounds and 68 hub targets were used to establish an interactive network of EU-RAB in the treatment of OA. As shown in Fig. 5, the network contained 103 nodes and 379 edges. Some compounds nodes were found to possess more edges, including quercetin (degree = 107), kaempferol (degree = 45), wogonin (degree = 17), baicalein (degree = 16), beta-sitosterol (degree = 17), etc., revealing that these compounds linked more targets and served a critical role in EU-RAB anti-OA.

#### Construction of a compound-target-pathway network of EU-RAB anti-OA

**Fig. 6:**
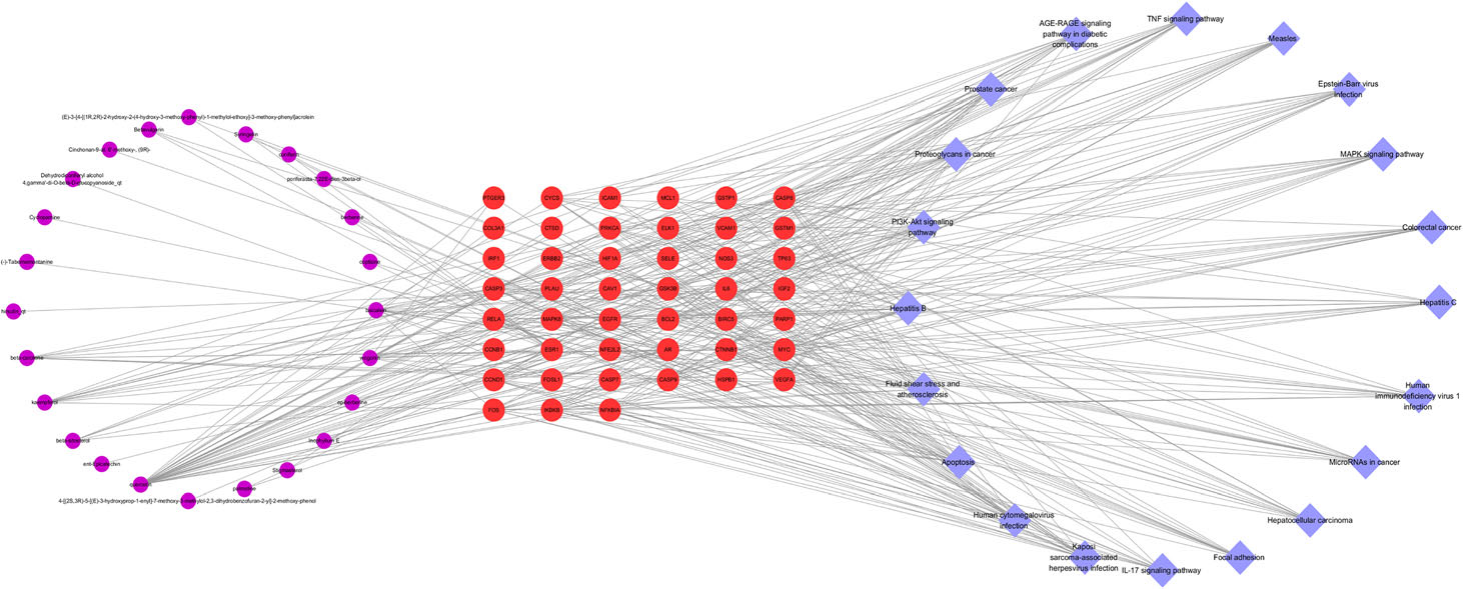
Compound-target-pathway network of *Eucommia ulmoides* (EU)-*Radix Achyranthis Bidentatae* (RAB) anti-Osteoarthritis (OA). The Compound-target-pathway network of EU-RAB anti-OA is constructed using 50 compounds, 68 co-targets, and 20 pathways, containing 89 nodes and 380 edges. Purple, red, and blue cycles represent compound, target, and pathway, respectively

To further reveal the mechanism action of EU-RAB on OA, a systematic and comprehensive bioinformatics network was constructed using 50 active compounds, 68 potential gene targets, and 20 KEGG signaling pathways. The complex network contained 89 nodes and 380 edges, indicating that there were complex interactions among multiple compounds-targets-pathways, which also played a synergistic effect on inhibiting the progression of OA. Some targets/pathways were also likely to play critical roles in osteoarthritis. In the bioinformatics network, associated signaling pathways including apoptosis (degree = 15), PI3K/Akt signaling pathway (degree = 15), Cysteinyl aspartate-specific proteinase 3 (Caspase3, degree = 21), inhibitor of nuclear factor kappa-B kinase subunit beta (IKBKB, degree = 16) and Glycogen Synthase Kinase 3 beta (GSK3β, degree = 16) were identified. The details of the compound-target-pathway network are shown in Table 2 and Figs. 3 and 6.

### PPI network construction

**Fig. 7:**
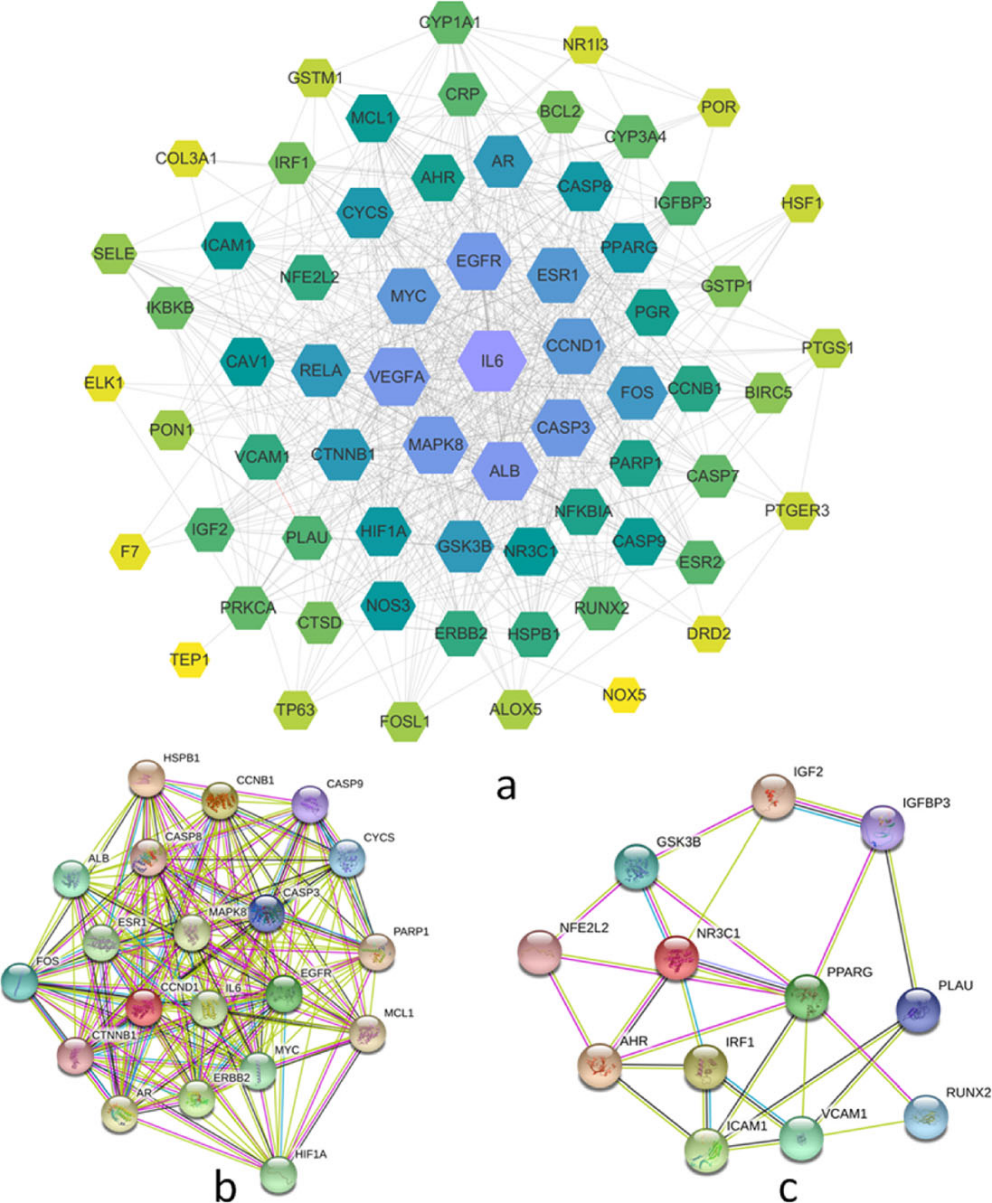
PPI network of *Eucommia ulmoides* (EU)-*Radix Achyranthis Bidentatae* (RAB)-Osteoarthritis (OA) genes. EU-RAB-OA genes PPI network (**a**) contains 68 nodes and 699 edges. Core genes have a higher degree and are positively correlated with node size and color depth, including, IL-6, VEGFA, EGFR, MAPK8, CASP3, MYC, CCND1, and ESR1. Two clusters are discovered in the PPI network. Cluster 1(**b**) includes 28 nodes and 290 edges, and Cluster 2(**c**) includes 12 nodes and 25 edges

A total of 68 hub gene targets were imported into STRING for the construction of a protein-protein interaction network. A PPI network containing 68 nodes and 699 edges (Fig. 7a) was obtained. The core gene targets possessed higher degree and were more likely to play a key role in EU-RAB anti-osteoarthritis including, Interleukin-6 (IL-6), Vascular endothelial growth factor (VEGFA), Estrogen receptor (ESR1), Epidermal growth factor receptor (EGFR), Mitogen-activated protein kinase 8 (MAPK8) and Caspase3.

MCODE analysis of PPI network based on the STRING database and Cytoscape software resulted in 2 Clusters networks (Fig. 7b, c and Supplementary file, Table S3). The higher score of modules was regarded as the more meaningful modules in the PPI network.

### Verification of compound-target interaction

**Fig. 8:**
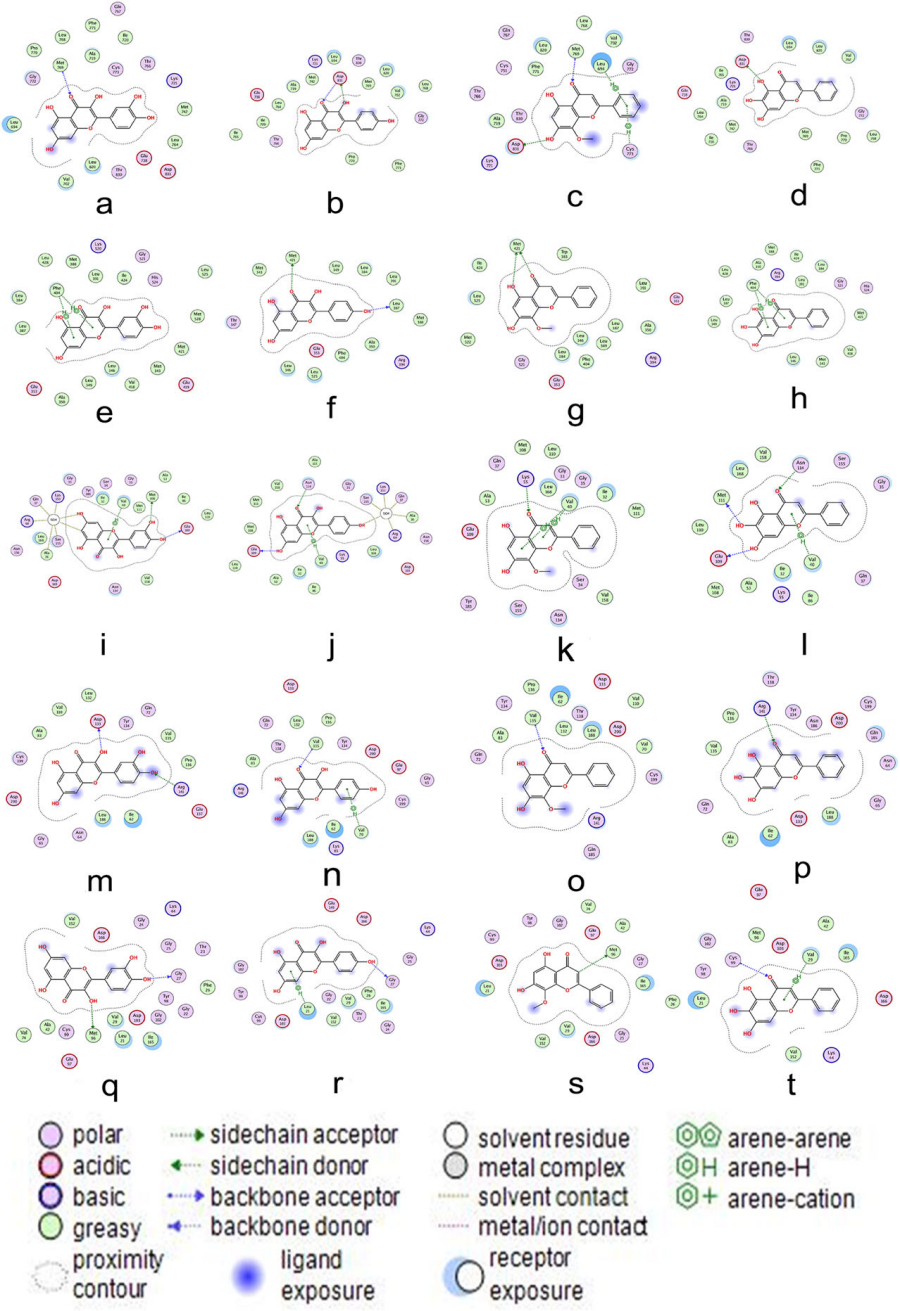
Virtual docking of bioactive ingredients from *Eucommia ulmoides*-*Radix Achyranthis Bidentatae* for Osteoarthritis targets. The molecular docking of quercetin, kaempferol, wogonin, and baicalein with EGFR (**a, b, c, d**), ESR1 (**e, f, g, h**), MAPK8 (**I, j, k, l**), GSK3β (**m, n, o, p**), and IKBKB (**q, r, s, t**) are shown

Due to lack of corresponding ligands of a few targets in the PDB, EGFR (PDB id: 1 m17), ESR1 (PDB id: 1r5k), MAPK8 (PDB id: 3pze), GSK3β (PDB id: 1q3d) and IKBKB (PDB id:4kik) were selected based on the core genes, signal pathway and research of osteoarthritis as successfully constructed proteins receptor for molecular docking verification. In molecular docking, if the ligand can bind to one or more amino acid residues with an H bond, H-π bond or π-π bond in the active site (also called active pocket) of the receptor and participates in the process of conformation change, energy complementation, etc., then the small molecule ligand can combine with the receptor to form a stable structure [28]. The binding atoms, binding sites, and binding energy intuitively show the interaction and stability of the docking model. In general, the stable docking model has negative binding energy, lower energy score, the stronger binding ability of ligand-receptor, and a more stable structure [29, 30]. In this study, each protein was successfully docked with 4 bioactive compounds (including quercetin, kaempferol, wogonin, baicalein) derived from the compound-target network, and acquired a stable docking model with a specific binding site, binding distance, and binding atom (Table 3 and Fig. 8). For example, Fig. 8a indicates that quercetin formed an H bond conjugation with Met 769 (3.23 Å) in EGFR. The ESR1-quercetin complex was stabilized in PHE 404 (4.18 Å, 3.95 Å) by two π-H bonds (Fig. 8e). Quercetin interacted with MAPK8 via five H bonds and one π-H bond on SO4 402 (3.06 Å), SO4 402 (3.17 Å), SO4 402 (3.48 Å), MET 108 (3.00 Å), GLU 109 (3.15 Å) and VAL 40 (3.87 Å) (Fig. 8i). The GSK3β-quercetin complex was connected to ASP 133 (2.97 Å) and ARG 141 (3.08 Å) by two π-H bonds (Fig. 8m). IKBKB and quercetin were bound in MET 96 (3.25 Å) and GLY 27 (2.76 Å) by two H bonds (Fig. 8q). Therefore, quercetin interacted stably with these targets through an H bond or π-H bond, and the specific action sites were Met 769, PHE 404, SO4 402, MET 108, GLU 109, VAL 40, ASP 133, ARG 141, MET 96 and GLY 27. Besides, compared with other targets, the docking energy of quercetin with IKBKB (− 7.1583 kcal·mol^− 1^) was the lowest, indicating that the binding ability and stability of quercetin-IKBKB complex was higher compared with the other targets.

## Discussion

Network pharmacology is a rational approach used to analyze the potential biological mechanism of TCM in the treatment and prevention of various diseases, and especially in chronic and frequently-occurring disease, such as osteoarthritis [20, 31]. Previous studies have confirmed that EU-RAB plays an important role in the treatment of OA [5, 6], but the mechanism of action is still not fully understood. Therefore, in the present study, for the first time, network pharmacology was performed to systematically uncover the mechanism of action of EU-RAB against OA. Moreover, the stable molecular docking model of the compound-target exhibited effective binding in representative compounds and hub OA targets, which further verified the inner links between EU-RAB and OA. These typical computer models/figures highlighted the direct effect of EU-RAB on OA, and also showed “multiply compounds, multiply targets, multiply pathways” potential mechanisms.

Through the screening of active ingredients and analysis of compound-target network, quercetin, kaempferol, wogonin, and baicalein were identified to be the most important active ingredients of EU-RAB in the treatment of OA. Previous studies have shown that quercetin promotes chondrocytes proliferation and acts on inflammatory injury to accelerate cartilage repair [32]. Increasingly evidence has associated kaempferol with the inflammation process. The anti-inflammation function of kaempferol is manifested in the modulation of cytokines and suppression of MAPK pathways [33]. Wogonin and baicalein are major flavones constituents with chondroprotective effects. Wogonin possesses a tight binding affinity with chondrocytes genomic DNA and stabilizes the DNA fragmentation to protect chondrocytes [34]. The anti-apoptosis and anti-catabolic effects of baicalein related to OA are embodied in an increase of glycosaminoglycan and type II collagen [35]. Notably, these active ingredients of EU-RAB synergistically exert anti-inflammation, anti-apoptosis, and maintenance of cartilage homeostasis effects, which can be regarded as potential therapeutic strategies against OA.

By integrating all the targets of EU-RAB and OA, 68 targets related to OA were identified. The 68 targets were used to construct PPI for use in highlighting pivotal targets and interaction degree with others, such as IL6, EGFR, VEGFA, CSAP3, ESR1, GSK3β, etc. In the entire target network of EU-RAB against OA, these targets showed a rich interaction with others and were more likely to produce a cascade effect for exerting antagonistic effects on OA. Moreover, in the C-T-P network, gene targets acting on multiple pathways at the same time were also found, indicating the importance of specific targets in the entire OA bioinformatics network. For instance, MAPK8 (degree = 14) appeared in apoptosis, TNF, and MAPK signaling pathways, and both PI3/Akt and IL-17 signaling pathways contained GSK3β (degree = 16). This also reflected the “multiply targets-multiply pathways” regulation mechanism of EU-RAB. IL-6 is a key pro-inflammatory mediator cytokine related to bone resorption and degradation of the extracellular matrix [36]. VEGFR serves as an important target participating in the mediation of inflammation, cell proliferation, and pain management [37, 38]. Caspase3 and GSK3β as the key apoptotic cytokines medicating DNA fragmentation and oxidant stress injury resulting in the acceleration of chondrocyte apoptosis [39, 40]. Therefore, the above targets were believed to be involved in the key molecular action site of EU-RAB anti-OA.

GO and KEGG analysis revealed that “apoptosis”, “TNF signal”, “IL-17 signal”, “PI3K/Akt signal pathway” and “MAPK signal pathway” were related to the mechanism of EU-RAB in the treatment of OA. In this study, “apoptosis” mapped the most key targets and possessed the highest degree of enrichment, and was the most dominant signaling way of EU-RAB against OA. Combined with hub targets of BP and MF analysis, the anti-apoptotic effect of EU-RAB reflected on the mediation of oxidative stress (GO:0006979, GO:0034599) and apoptotic signaling (GO:2001233, GO:0097153). Besides, the active ingredients of EU-RAB were found to possess a good anti-apoptosis effect via inhibition of inflammatory induction or directly suppressing cellular stress state [32–35]. Simultaneously, molecular docking results showed that a representative apoptotic cytokine, GSK3β had a good binding affinity with the above active ingredients, which also confirmed the direct anti-apoptotic effect of EU-RAB. Aberrant apoptosis resulted in a decrease of chondrocytes number and abnormal morphology and function, which led to difficulties in sustaining the mechanical stress balance of the extracellular matrix as well as induce cartilage matrix degradation [41, 42]. Furthermore, accompanying disorders of physicochemical factors or inflammatory cytokines during aberrant apoptosis produced a high concentration of reactive oxygen species leading to oxidative stress which disturbed the tranquility of cartilage homeostasis [43, 44]. Inflammatory cascade, apoptotic protein, and NF-Kβ signaling are the main promoters of Tumor Necrosis Factor (TNF) driving the multiply biological function, especially the inflammatory and apoptotic response [45]. In this study, EU-RAB was found to inhibit the activity of TNF signaling by affecting 15 targets such as IL6, IKBKB, Caspase3, etc. Besides, as an important member of the NF-Kβ family, IKBKB formed a good visualization model with the main active ingredients in molecular docking analysis. These results showed the good target effect of EU-RAB on TNF signaling. Interleukin-17 (IL-17) signaling played a key role in the pathophysiological process of Immunomodulation and infection, osteoarthritis susceptibility [46], and pain control [47]. MAPK is a member of the serine-threonine protein kinases family and a transduction center for cell multidirectional changes involved in the development of OA [48]. PI3K/AKT signaling, is an important signal transduction site associated with the pathophysiological process of intracellular metabolism, including cell proliferation, autophagy, and apoptosis [49].

In this study, network pharmacology was used to elaborate on the potential anti-osteoarthritis effect of EU-RAB and this effect was visualized by molecular docking for verification. This scientific and systematic theoretical study provides a basis for future pharmacological studies to further explore the mechanism of EU-RAB in the treatment of OA.

## Conclusion

Traditional Chinese medicine has a long history for thousands of years in the treatment of osteoarthritis. As a typical representative, EU-RAB has been reported to play a protective effect through multiple targets and pathways in OA. For the first time, this study systematically expounds on the mechanism of action of EU-RAB anti-OA using network pharmacology. Besides, this effect is visualized through molecular docking to verify this relationship. In this study, 50 main bioactive compounds of EU-RAB anti-OA are identified. Moreover, IL-6, EGFR, VEGFA, ESR1, CASP3, GSK3β, MAPK8, etc., are the pivotal genes enriched to signaling transduction pathways (e.g apoptosis, TNF signaling) and for EU-RAB to exert its anti-apoptosis and anti-inflammatory effect on OA. Therefore, the analysis of molecular biological networks based on multiply target-pathway interaction using network pharmacology not only contributes to deepening the understanding of EU-RAB against OA but also provides a pharmacology basis for further research.

## Supplementary information

**Additional file 1: Table S1**. Active compounds of *Eucommia ulmoides* (EU)-*Radix Achyranthis Bidentatae* (RAB). **Table S2.** Potential targets of *Eucommia ulmoides* (EU)-*Radix Achyranthis* *Bidentatae* (RAB). **Table S3.** Cluster network of target genes of *Eucommia ulmoides* (EU)-*Radix Achyranthis* *Bidentatae* (RAB) against Osteoarthritis (OA).

## Data Availability

The data used to support the findings of this study are available from the corresponding author upon request, unless there are legal or ethical reasons for not doing so.

## Abbreviations

OA: Osteoarthritis
TCM: Traditional Chinese Medicine
EU: *Eucommia ulmoides*
RAB: *Radix Achyranthis Bidentatae*
TCMSP: Traditional Chinese Medicine Systems Pharmacology
BATMAN-TCM: Bioinformatics Analysis Tool for Molecular mechanism of Traditional Chinese Medicine
OB: Oral Bioavailability
DL: Druglikeness
ADME: Absorption, Distribution, Metabolism and Excretion
GO: Gene ontology
KEGG: Kyoto Encyclopedia of Genes and Genomes
PPI: Protein-protein interaction
BP: Biological Processes
CC: Cell Components
MF: Molecular Functions
Caspase3: Cysteinyl aspartate-speciwc proteinase 3
GSK3ß: Glycogen Synthase Kinase 3 beta
IKBKB: Inhibitor of nuclear factor kappa-B kinase subunit beta
ESR1: Estrogen receptor
EGFR: Epidermal growth factor receptor
MAPK8: mitogen-activated protein kinase
VEGF: Vascular endothelial growth factor
IL-6: Interleukin-6
TNF: Tumor Necrosis Factor
IL-17: Interleukin-17
