# Supplementary material for: Application of network pharmacology and molecular docking to elucidate the potential mechanism of Eucommia ulmoides-Radix Achyranthis Bidentatae against osteoarthritis
Source: BioData Min. 2020 Aug 28;13:12. doi: 10.1186/s13040-020-00221-y (PMC7456016; doi:10.1186/s13040-020-00221-y)
Supplement: Supplementary file 1 — Additional file 1: Table S1. Active compounds of Eucommia ulmoides (EU)-Radix Achyranthis Bidentatae (RAB). Table S2. Potential targets of Eucommia ulmoides (EU)-Radix Achyranthis Bidentatae (RAB). Table S3. Cluster network of target genes of Eucommia ulmoides (EU)-Radix Achyranthis Bidentatae (RAB) against Osteoarthritis (OA). [file 13040_2020_221_MOESM1_ESM.docx]

**Table S1 Active compounds of Eucommia ulmoides (EU)-Radix Achyranthis (RAB)**

| Molecular ID | Compound | MW | OB (%) | DL | | Herb | Database source |
| --- | --- | --- | --- | --- | --- | --- | --- |
| MOL000098 | quercetin | 302.25 | 46.43 | 0.28 | EU,RAB | | TCMSP |
| MOL000358 | beta-sitosterol | 414.79 | 36.91 | 0.75 | EU,RAB | | TCMSP |
| MOL000422 | kaempferol | 286.25 | 41.88 | 0.24 | EU,RAB | | TCMSP,BATMAN-TCM |
| MOL000073 | ent-Epicatechin | 290.29 | 48.96 | 0.24 | EU | | TCMSP |
| MOL000211 | Mairin | 456.78 | 55.38 | 0.78 | EU | | TCMSP |
| MOL000443 | Erythraline | 297.38 | 49.18 | 0.55 | EU | | TCMSP,BATMAN-TCM |
| MOL002058 | 40957-99-1 | 388.45 | 57.2 | 0.62 | EU | | TCMSP |
| MOL002773 | beta-carotene | 536.96 | 37.18 | 0.58 | EU | | TCMSP |
| MOL004367 | olivil | 376.44 | 62.23 | 0.41 | EU | | TCMSP |
| MOL005922 | Acanthoside B | 580.64 | 43.35 | 0.77 | EU | | TCMSP,BATMAN-TCM |
| MOL006709 | AIDS214634 | 374.42 | 92.43 | 0.55 | EU | | TCMSP |
| MOL007059 | 3-beta-Hydroxymethyllenetanshiquinone | 294.32 | 32.16 | 0.41 | EU | | TCMSP |
| MOL007563 | Yangambin | 446.54 | 57.53 | 0.81 | EU | | TCMSP |
| MOL008240 | (E)-3-[4-[(1R,2R)-2-hydroxy-2-(4-hydroxy-3-methoxy-phenyl)-1-methylol-ethoxy]-3-methoxy-phenyl]acrolein | 374.42 | 56.32 | 0.36 | EU | | TCMSP |
| MOL009007 | Eucommin A | 550.61 | 30.51 | 0.85 | EU | | TCMSP |
| MOL009009 | (+)-medioresinol | 388.45 | 87.19 | 0.62 | EU | | TCMSP |
| MOL009015 | (-)-Tabernemontanine | 354.49 | 58.67 | 0.61 | EU | | TCMSP |
| MOL009027 | Cyclopamine | 411.69 | 55.42 | 0.82 | EU | | TCMSP,BATMAN-TCM |
| MOL009029 | Dehydrodiconiferyl alcohol 4,gamma'-di-O-beta-D-glucopyanoside_qt | 358.42 | 51.44 | 0.4 | EU | | TCMSP |
| MOL009030 | Dehydrodieugenol | 326.42 | 30.1 | 0.24 | EU | | TCMSP,BATMAN-TCM |
| MOL009031 | Cinchonan-9-al, 6'-methoxy-, (9R)- | 324.46 | 68.22 | 0.4 | EU | | TCMSP |
| MOL009038 | GBGB | 550.57 | 45.58 | 0.83 | EU | | TCMSP |
| MOL009042 | Helenalin | 262.33 | 77.01 | 0.19 | EU | | TCMSP, BATMAN-TCM |
| MOL009047 | (+)-Eudesmin | 386.48 | 33.29 | 0.62 | EU | | TCMSP |
| MOL009053 | 4-[(2S,3R)-5-[(E)-3-hydroxyprop-1-enyl]-7-methoxy-3-methylol-2,3-dihydrobenzofuran-2-yl]-2-methoxy-phenol | 358.42 | 50.76 | 0.39 | EU | | TCMSP |
| MOL009055 | hirsutin_qt | 345.35 | 49.81 | 0.37 | EU | | TCMSP,BATMAN-TCM |
| MOL009057 | liriodendrin_qt | 450.48 | 53.14 | 0.8 | EU | | TCMSP |
| MOL011604 | Syringetin | 346.31 | 36.82 | 0.37 | EU | | TCMSP |
| MOL013440 | Citrusin B | 568.63 | 40.80 | 0.71 | EU | | BATMAN-TCM |
| MOL002813 | Aucubin | 346.37 | 35.56 | 0.33 | EU | | BATMAN-TCM |
| MOL000519 | Coniferin | 314.41 | 31.11 | 0.32 | EU | | BATMAN-TCM |
| MOL001006 | poriferasta-7,22E-dien-3beta-ol | 412.77 | 42.98 | 0.76 | RAB | | BATMAN-TCM |
| MOL012461 | 28-norolean-17-en-3-ol | 412.77 | 35.93 | 0.78 | RAB | | TCMSP |
| MOL012505 | bidentatoside,ii_qt | 556.86 | 31.76 | 0.59 | RAB | | TCMSP |
| MOL012537 | Spinoside A | 716.95 | 41.75 | 0.4 | RAB | | TCMSP |
| MOL012542 | β-ecdysterone | 480.71 | 44.23 | 0.82 | RAB | | TCMSP |
| MOL001454 | berberine | 336.39 | 36.86 | 0.78 | RAB | | TCMSP |
| MOL001458 | coptisine | 320.34 | 30.67 | 0.86 | RAB | | TCMSP |
| MOL000173 | wogonin | 284.28 | 30.68 | 0.23 | RAB | | TCMSP |
| MOL002643 | delta 7-stigmastenol | 414.79 | 37.42 | 0.75 | RAB | | TCMSP |
| MOL002714 | baicalein | 270.25 | 33.52 | 0.21 | RAB | | TCMSP,BATMAN-TCM |
| MOL002776 | Baicalin | 446.39 | 40.12 | 0.75 | RAB | | TCMSP |
| MOL002897 | epiberberine | 336.39 | 43.09 | 0.78 | RAB | | TCMSP |
| MOL003847 | Inophyllum E | 402.47 | 38.81 | 0.85 | RAB | | TCMSP |
| MOL004355 | Spinasterol | 412.77 | 42.98 | 0.76 | RAB | | TCMSP |
| MOL000449 | Stigmasterol | 412.77 | 43.83 | 0.76 | RAB | | TCMSP |
| MOL000785 | palmatine | 352.44 | 64.6 | 0.65 | RAB | | TCMSP |
| MOL000085 | beta-daucosterol_qt | 414.79 | 36.91 | 0.75 | RAB | | TCMSP |
| MOL012286 | Betavulgarin | 312.29 | 68.75 | 0.39 | RAB | | TCMSP |
| MOL012298 | Rubrosterone | 334.45 | 32.69 | 0.47 | RAB | | TCMSP,BATMAN-TCM |

**Table S2 Potential targets of Eucommia ulmoides (EU)-Radix Achyranthis (RAB)**

| N0. | Gene | N0 | Gene | N0 | Gene | N0 | Gene | N0 | Gene | |
| --- | --- | --- | --- | --- | --- | --- | --- | --- | --- | --- |
| 1 | PTGS1 | 23 | TP63 | 45 | HSPB1 | 67 | IGF2 | 89 | ALB |  |
| 2 | AR | 24 | ELK1 | 46 | MGAM | 68 | IRF1 | 90 | CTNNB1 |  |
| 3 | PPARG | 25 | NFKBIA | 47 | CYP1B1 | 69 | ERBB3 | 91 | CASP7 |  |
| 4 | NCOA2 | 26 | POR | 48 | CCNB1 | 70 | PON1 | 92 | NCOA1 |  |
| 5 | AKR1B1 | 27 | CASP8 | 49 | ALOX5 | 71 | DIO1 | 93 | GSK3B |  |
| 6 | PRSS1 | 28 | RAF1 | 50 | GSTP1 | 72 | NPEPPS | 94 | HTR3A |  |
| 7 | F7 | 29 | PRKCA | 51 | NFE2L2 | 73 | HK2 | 95 | DRD3 |  |
| 8 | ACHE | 30 | HIF1A | 52 | NQO1 | 74 | RASA1 | 96 | DRD2 |  |
| 9 | GABRA1 | 31 | RUNX1T1 | 53 | PARP1 | 75 | GSTM1 | 97 | PDE10A |  |
| 10 | RELA | 32 | ERBB2 | 54 | AHR | 76 | GSTM2 | 98 | NR3C2 |  |
| 11 | EGFR | 33 | ACACA | 55 | PSMD3 | 77 | PGR | 99 | NR3C1 |  |
| 12 | VEGFA | 34 | CYP3A4 | 56 | SLC2A4 | 78 | ESR1 | 100 | ADRA2C |  |
| 13 | CCND1 | 35 | CAV1 | 57 | COL3A1 | 79 | CHRM3 | 101 | DRD4 |  |
| 14 | BCL2 | 36 | MYC | 58 | DCAF5 | 80 | CHRM1 | 102 | ESR2 |  |
| 15 | FOS | 37 | CYP1A1 | 59 | NR1I3 | 81 | CHRM4 | 103 | FOSL1 |  |
| 16 | EIF6 | 38 | ICAM1 | 60 | CHEK2 | 82 | ADRA1A | 104 | CYCS |  |
| 17 | CASP9 | 39 | SELE | 61 | HSF1 | 83 | CHRM2 | 105 | NOX5 |  |
| 18 | PLAU | 40 | VCAM1 | 62 | CRP | 84 | CHRNA2 | 106 | APOD |  |
| 19 | RB1 | 41 | PTGER3 | 63 | RUNX2 | 85 | IKBKB | 107 | CHEK1 |  |
| 20 | IL6 | 42 | BIRC5 | 64 | RASSF1 | 86 | MAPK8 | 108 | TEP1 |  |
| 21 | AHSA1 | 43 | DUOX2 | 65 | CTSD | 87 | AKR1C3 | 109 | MCL1 |  |
| 22 | CASP3 | 44 | NOS3 | 66 | IGFBP3 | 88 | CHRM5 | 110 | CTRB1 |  |

**Table S3 Cluster network of target genes of Eucommia ulmoides (EU)-Radix Achyranthis (RAB) against Osteoarthritis (OA)**

| Cluster | Score | Nodes | Edges | Node IDs |
| --- | --- | --- | --- | --- |
| 1 | 21.091 | 28 | 290 | ALB, AR, CASP3, CASP8, CASP9, CCNB1, CCND1,CTNNB1, CYCS, EGFR, ERBB2, ESR1, FOS, HIF1A,HSPB1, IL6, MAPK8, MCL1, MYC, PARP1, PGR, RELA,SERPINE1, CRP, MMP3, SPP1, IL1A, CXCL8 |
| 2 | 4.5 | 12 | 25 | IGF2, RUNX2, VCAM1, NR3C1, AHR, GSK3B, PPARG,, ICAM1, NFE2L2, PLAU, IRF1, IGFBP3 |
